# Cellular senescence in ovarian cancer: context-dependent effects on tumor immunity and therapy response

**DOI:** 10.3389/fimmu.2026.1965539

**Published:** 2026-10-26

**Authors:** Yubo Zhang, Mengqi Deng, Junyi Jiang

**Affiliations:** 1 Qingdao Hospital, University of Health and Rehabilitation Sciences (Qingdao Municipal Hospital), Qingdao, China; 2 Beijing Obstetrics and Gynecology Hospital, Capital Medical University, Beijing Maternal and Child Health Care Hospital, Beijing, China; 3 Laboratory for Clinical Medicine, Capital Medical University, Beijing, China; 4 State Key Laboratory of Medical Proteomics, National Center for Protein Sciences (Beijing), Beijing, China

**Keywords:** cellular senescence, high-grade serous ovarian cancer, immunosenolysis, ovarian cancer, platinum resistance, therapy-induced senescence, tumor immunity, tumor microenvironment

## Abstract

Cellular senescence is a stress-responsive state marked by sustained proliferative arrest and changes in metabolism, chromatin organization, secretion, and immune signaling. In ovarian cancer (OC), platinum therapy and PARP inhibition can generate senescence-associated states with distinct consequences. Their effects depend on treatment context, cellular compartment, stability of arrest, and the surrounding immune state. Therapy-induced senescent tumor cells may increase antigen presentation and inflammatory signaling and, in immune-competent settings, facilitate dendritic-cell and CD8+ T-cell responses. Persistent senescence can instead maintain a SASP that remodels stromal and immune compartments and may support residual disease, dissemination, or relapse. OC studies have linked altered senescence responses to cisplatin resistance, while senescence-associated states have also been reported after PARP inhibition and taxane treatment. Senescence in CAFs, mesothelial cells, adipose-associated stroma, and immune cells can further alter tumor-cell survival, invasion, immune surveillance, and treatment response. We therefore distinguish stable TIS from reversible senescence-like or dormant states and separate tumor-cell effects from stromal and immune effects. Senolysis, SASP modulation, ferroptosis, and immune-mediated clearance represent potential strategies, but most evidence remains preclinical. Overall, senescence in OC is best viewed as a context-dependent state that can either support antitumor immunity or facilitate immune escape and treatment persistence.

## 1 Introduction

Ovarian cancer (OC) remains a major cause of cancer-related mortality among women (1). OC comprises several histopathological and molecular subtypes, including high-grade serous, low-grade serous, endometrioid, clear cell, and mucinous carcinomas, which differ in their cellular origins, molecular alterations, and clinical behavior (2, 3). High-grade serous ovarian cancer (HGSOC) is the predominant epithelial subtype and accounts for a large proportion of OC-related deaths (3).

Platinum-based chemotherapy remains a central component of treatment for advanced OC. Although many patients initially respond, recurrence is common and a substantial proportion eventually develop platinum-resistant disease (4). Platinum sensitivity and platinum resistance should nevertheless be distinguished from the broader concept of recurrent disease. Patients with platinum-sensitive recurrence may derive clinically meaningful benefit from subsequent platinum-containing therapy, whereas platinum-resistant disease is characterized by limited benefit from further platinum treatment (4). The mechanisms underlying platinum resistance are heterogeneous and include alterations in DNA damage repair, drug transport and detoxification, apoptosis, epigenetic regulation, redox homeostasis, and interactions with the tumor microenvironment (TME) (5).

Cellular senescence is a stress-associated state characterized by sustained proliferative arrest accompanied by changes in cellular morphology, metabolism, chromatin organization, and secretory activity (6, 7). Senescence can arise in response to telomere dysfunction, DNA damage, oncogenic signaling, oxidative stress, and anticancer treatment (8, 9). Senescent cells may develop a senescence-associated secretory phenotype (SASP) comprising cytokines, chemokines, growth factors, proteases, and other extracellular factors. The SASP is heterogeneous rather than a fixed molecular program; its composition depends on the inducing stimulus, cell type, duration of senescence, and tissue environment (8).

A key reason for this context dependence is that senescence can be both immunogenic and tumor-promoting. Senescent cells can increase antigen presentation and type I interferon-associated signaling and promote dendritic-cell and CD8+ T-cell activation, providing a plausible route to immune-mediated tumor control; however, these observations derive largely from non-ovarian models and should be interpreted as a conserved mechanism rather than OC-specific clinical evidence (10). In parallel, therapy-induced senescent tumor cells may persist in a dormant or reversible state and later contribute to relapse, including through stemness-associated reprogramming (11–13).

In cancer therapy, senescence occupies an ambiguous position. Treatment-induced growth arrest may contribute to tumor control by preventing proliferation and, in some settings, facilitating immune recognition and clearance. However, cells that survive treatment in a senescence-associated state may retain the capacity for phenotypic adaptation, and some senescence-associated programs can alter the surrounding microenvironment in ways that favor tumor persistence or progression. Importantly, the presence of a senescence-associated marker does not by itself demonstrate functional senescence, and the presence of senescence does not by itself establish treatment resistance.

This distinction is particularly important in OC, where evidence has accumulated from different therapeutic settings. Cisplatin and other platinum compounds have been studied in relation to senescence, but other studies have examined PARP inhibitors, paclitaxel, or docetaxel. These treatments impose different biological pressures and should not be assumed to produce equivalent resistance states. Similarly, senescence in malignant epithelial cells is biologically distinct from senescence in cancer-associated fibroblasts (CAFs), mesothelial cells, adipose-associated stromal populations, or immune cells.

This review focuses on how therapy-associated senescence shapes treatment response in ovarian cancer, with emphasis on platinum therapy and its interactions with tumor cells, stromal compartments, and immune surveillance. We distinguish direct evidence for platinum resistance from findings that concern other treatments or disease progression.

From an immunological perspective, the key question is whether a treatment-induced senescence state can be recognized and cleared or instead becomes permissive for immune escape. The same TIS program may increase antigen presentation and interferon-associated signaling while also inducing checkpoint protection or chronic SASP activity. Its effects therefore depend on antigen-presenting cell competence, T- and NK-cell function, stromal context, and the duration of exposure (10, 14–17).

## 2 Therapy-induced senescence in ovarian cancer: induction and tumor-suppressive effects

Platinum compounds induce DNA damage and replication-associated stress, which can activate cell-cycle checkpoints and either promote cell death or establish a senescence-associated growth-arrest program (9, 18). The outcome is influenced by the extent and duration of DNA damage, intracellular drug exposure, DNA repair capacity, genetic background, and the integrity of pathways regulating cell-cycle arrest and apoptosis.

Two canonical cell-cycle pathways help determine whether treatment-induced stress becomes a stable arrest. DNA-damage signaling can activate p53 and p21, which inhibit cyclin-dependent kinases and reinforce RB-mediated repression of E2F. The p16INK4a–RB pathway provides a complementary brake on CDK4/6 activity. In ovarian cancer, olaparib-induced senescence has been linked to p16- or p53-dependent RB signaling, although the relative contribution of these pathways varies across models (9, 19).

Apoptosis and senescence should therefore not be regarded as mutually exclusive or predetermined outcomes of platinum treatment. At sufficiently high levels of cellular injury, apoptosis may dominate. Under conditions in which stress is substantial but not immediately lethal, cells may enter a prolonged growth-arrested state. Experimental studies in OC have reported senescence-associated phenotypes after platinum treatment and other anticancer therapies (18, 20).

Evidence from HGSOC models supports the existence of treatment-associated senescence programs but also illustrates their context dependence. Treatment of HGSOC models with cisplatin or the RNA polymerase I inhibitor CX-5461 has been associated with senescence-related transcriptional and phenotypic changes (21). These findings indicate that therapy-associated senescence can be detected in HGSOC, but they do not establish that all senescent cells share the same molecular program or that senescence is synonymous with platinum resistance.

Importantly, senescence can contribute to tumor control. Platinum-based chemotherapy has been reported to induce senescence-associated inflammatory remodeling of the ovarian carcinoma microenvironment (22). In preclinical OC models, the extent of senescence induction influenced responses to chemotherapy and immunotherapy, indicating that treatment-associated senescence can modify antitumor responses (23). These observations support a tumor-suppressive role for senescence under defined conditions, although the magnitude and durability of this effect depend on the model and treatment context.

An important counterpoint is that senescence is not intrinsically synonymous with an immunosuppressive state. Marin et al. showed across fibroblast and cancer-cell models that therapy-induced senescence can increase MHC class I antigen-presentation machinery, activate interferon signaling, and enhance dendritic-cell and antigen-specific CD8+ T-cell responses (10). These findings provide a mechanistic basis for increased immune visibility, but immune visibility does not guarantee immune clearance. Hwang et al. found that therapy-induced senescent cancer cells can also upregulate PD-L1 through RPN1-dependent mechanisms, reducing susceptibility to T-cell-mediated killing. RPN1 depletion or PD-1 blockade enhanced elimination of senescent cells in preclinical models (15). Because these studies were not conducted as OC-specific clinical trials, they provide mechanistic context rather than direct clinical evidence for OC.

Importantly, this immunological model is supported by primary evidence in HGSOC. Using an immunocompetent autochthonous ovarian cancer model, Paffenholz et al. showed that the capacity of HR-deficient tumors to undergo therapy-induced senescence contributed to platinum sensitivity, and that a restricted cGAS/STING-dependent SASP was sufficient to promote immune infiltration and increase sensitivity to immune checkpoint blockade (23). This study therefore connects senescence, genotype-dependent treatment response, and immune activation within an ovarian cancer context. It also illustrates an important boundary of the evidence: the beneficial effect depended on a defined senescence program and an immune-competent host, rather than on senescence markers alone.

An immunogenic TIS response, however, depends on more than the phenotype of the senescent tumor cell itself. Antigen release or enhanced antigen presentation must be coupled to competent antigen-presenting cells, lymphocyte trafficking, and effector function. The simultaneous induction of MHC-I and immune-evasion pathways such as PD-L1 illustrates why “immunogenic senescence” should not be equated with automatic immune clearance (10, 15, 16). In the setting of treatment, these requirements may be undermined by metabolic stress, suppressive myeloid populations, or dysfunctional T and NK cells. Thus, a chemotherapy-induced increase in immune visibility does not necessarily translate into durable clearance; the outcome can diverge according to the integrity of the DC–T-cell axis, NK-cell surveillance, checkpoint signaling, and the suppressive tone of the TME (24, 25).

Thus, senescence should initially be viewed as one possible consequence of treatment-induced cellular stress rather than as a predefined mechanism of either treatment response or treatment failure (**Figure 1**).

**Figure 1:**
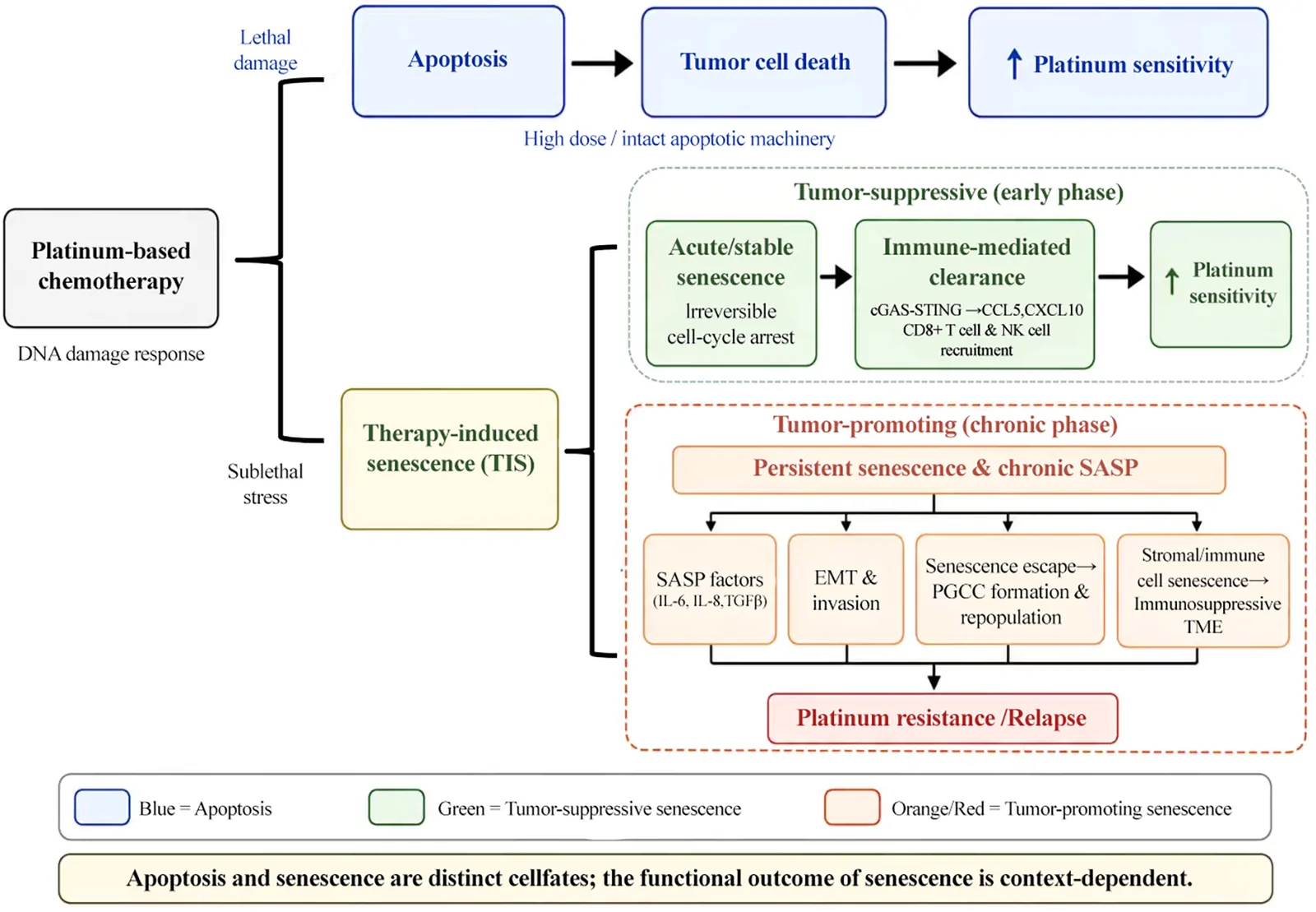
Therapy-induced senescence and immune context determine ovarian cancer cell fate. Platinum-based therapy can lead to apoptosis, persistent TIS, or reversible senescence-like states. Stable TIS may permit immune recognition and clearance, whereas chronic SASP and immune dysfunction may favor persistence and relapse. Reversible states may also be associated with plasticity and stemness in selected models (10, 15, 23, 26–29).

## 3 Persistent senescence, senescence escape, and cellular plasticity

The biological significance of treatment-associated senescence depends not only on whether cells stop proliferating, but also on how stable that arrest is. Some senescence-associated states are relatively persistent, whereas others can be reversed after withdrawal of the inducing stimulus (26) (**Table 1**). This distinction is relevant to the biology of residual disease because non-proliferating cells may survive cytotoxic treatment and subsequently re-enter the cell cycle or acquire alternative phenotypes.

**Table 1:** Evidence linking senescence-associated states to treatment response and disease progression in ovarian cancer.

Cellular compartment	Treatment/exposure	Senescence-associated state	Representative pathway/factor	Main reported outcome	Relationship to platinum resistance
Tumor cells	Cisplatin	Senescence suppression	Aurora-A/SOX8/FOXK1	Chemoresistance	Direct cisplatin-associated evidence (30)
Tumor cells	Cisplatin	Altered senescence response	NACC1/GADD45GIP1	Cisplatin resistance	Direct cisplatin-associated evidence (31)
Tumor cells	Cisplatin	Senescence-associated phenotype	GRP78/p53/CDC2	Modulation of cisplatin-induced senescence	Cisplatin-associated, model specific (32)
Tumor cells	Cisplatin	TIS-associated stem-like state	NAMPT	Persistence of cancer stem-like population	Platinum-associated evidence (33)
Tumor cells	Platinum treatment	Senescence-associated inflammatory response	cGAS-related signaling	Immune/TME remodeling	Platinum-associated evidence in immunocompetent OC models (23)
Tumor cells	PARP inhibitor	Reversible senescence-like TIS	BCL-XL-dependent apoptosis resistance	Senolytic targeting by ABT-263; reduced sensitivity after state reversal	PARP inhibitor evidence; not direct platinum evidence (26, 34)
Tumor cells	Platinum treatment	TGFβ-enriched senescent secretome	TGFBR1–AKT/mTOR	Tumor-promoting effects and treatment resistance	Cross-cancer mechanistic evidence; HGSOC translational evidence (28)
Tumor cells	PARP inhibitor	Senescence-associated state	PART1/PHB2	PARP inhibitor resistance	Not direct platinum evidence (35)
Tumor cells	Paclitaxel	Senescence-associated state	miR-433	Paclitaxel resistance	Not platinum evidence (36)
Tumor cells	Docetaxel	PGCC-associated senescence	OCT4/KLF4-associated plasticity	Stem-like phenotype	Not platinum evidence (27)
Tumor cells	Cisplatin	Senescence-associated iron dysregulation	Iron/ferroptosis	Vulnerability to ferroptosis	Platinum-associated therapeutic evidence (21)
CAFs	PARP inhibitor	Senescence/SASP	NF-κB	PARP inhibitor resistance	Not direct platinum evidence (37)
Mesothelial cells	Tumor-associated stress	Senescent mesothelial state	—	Ovarian cancer colonization	Metastasis evidence (38, 39)
Adipose-associated stroma	Carboplatin/olaparib	Senescence-associated state	—	Reduced metastasis after senolytic treatment	Metastasis-associated, not direct tumor-cell resistance (40)
Stromal/immune compartments	Treatment-associated stress	Senescence-associated signaling	WNT16B/extracellular vesicles	Immune or intercellular remodeling	Related evidence; not direct platinum resistance (41, 42)

CAF, cancer-associated fibroblast; OC, ovarian cancer; PGCC, polyploid giant cancer cell; SASP, senescence-associated secretory phenotype; TIS, therapy-induced senescence.

The distinction between senescence and dormancy is particularly important for relapse. Song et al. synthesized evidence that therapy-induced senescent tumor cells can contribute to local or metastatic relapse through persistent secretory signaling, phenotypic plasticity, and the emergence of relapse-competent populations (12). A Cancer Research report likewise proposed that tumor cells surviving therapy-induced senescence may represent a form of tumor dormancy from which rare cells recover self-renewal and proliferative capacity (13). These concepts suggest a continuum rather than a binary trajectory: a residual population may contain deeply arrested cells destined for clearance, reversible senescence-like cells capable of re-entry, and cells that acquire stem-like or drug-tolerant features. In OC, the NAMPT-dependent cancer stem-like population described after platinum treatment provides a disease-specific example of this broader model (33). Importantly, these studies do not establish that every senescence-associated residual cell becomes a CSC or that senescence itself is the initiating cause of relapse; they instead support testing the stability and functional consequences of the arrested state over time.

Senescence-associated reprogramming can also be accompanied by increased cellular plasticity and stem-like properties (11). In OC, metabolic and senescence-associated features have been linked to the immune microenvironment, treatment-selected resistant populations, and patient-derived organoid models (43–45). However, these observations do not establish that senescence itself is the initiating cause of resistance. In many models, senescence is better considered one component of a broader drug-adaptation program.

### 3.1 Suppression of senescence in cisplatin resistance

Several studies have linked impaired senescence responses to cisplatin resistance in defined OC models. Aurora-A/SOX8/FOXK1 signaling was reported to suppress cellular senescence and promote chemoresistance in ovarian cancer organoids and cell models (30). The resistant phenotype was also associated with increased glycolytic activity, although the study does not establish that glycolytic reprogramming alone is responsible for suppression of senescence.

NACC1 provides another example. The NACC1/GADD45GIP1 axis was reported to mediate cisplatin resistance through regulation of cellular senescence in ovarian cancer models (31). Together, these studies support the concept that failure to establish or maintain treatment-associated growth arrest can favor survival after cisplatin exposure in selected tumor-cell contexts.

These findings, however, should not be generalized into a universal model in which senescence suppression is required for platinum resistance. The underlying pathways may be particularly relevant to specific genetic and cellular backgrounds.

### 3.2 Senescence-associated states in other forms of treatment resistance

Other OC studies illustrate the opposite relationship: a senescence-associated phenotype may coexist with treatment resistance.

The NF-κB–HOTAIR axis has been associated with DNA damage responses, chemoresistance, and cellular senescence in OC (46). In another study, blockade of the lncRNA-PART1–PHB2 axis promoted cellular senescence while conferring resistance to PARP inhibition (35). The latter finding is important but does not constitute direct evidence of platinum resistance, because the treatment endpoint was PARP inhibitor response.

Similarly, miR-433 overexpression was reported to promote paclitaxel resistance through induction of a senescence-associated phenotype in OC cells (36). This provides evidence for a relationship between senescence-associated growth arrest and taxane resistance, rather than demonstrating a platinum-specific mechanism.

Saleh et al. proposed that prolonged senescence-like arrest can function as a form of tumor dormancy, from which a small subpopulation may recover self-renewal and contribute to recurrence (13). This is a conceptual model rather than evidence that all TIS is reversible, but it provides a useful framework for distinguishing durable arrest from relapse-competent residual cells.

An AACR 2017 Cancer Research abstract likewise reported transient therapy-induced senescence and subsequent proliferative recovery in an NSCLC model, supporting the possibility that a fraction of therapy-exposed tumor cells can exit a senescence-associated arrest (47). Because this evidence is conference-level and derived from NSCLC rather than OC, it is considered supporting context rather than disease-specific evidence.

A useful distinction is therefore between resistance caused by avoiding senescence and resistance that emerges after entering a senescence-associated state. In the first scenario, impaired growth arrest allows continued proliferation under treatment; in the second, growth arrest temporarily protects a residual population and creates time for metabolic, transcriptional, or stemness-related adaptation. The two mechanisms can coexist within the same tumor, particularly when genetically heterogeneous subclones experience different levels of DNA damage. This heterogeneity may explain why bulk measurements of “senescence” can obscure the behavior of the rare cells that ultimately seed recurrence.

These studies therefore argue against a simple linear model of “senescence equals sensitivity” or “senescence equals resistance.” Instead, the functional consequence depends on the treatment, molecular context, and properties of the surviving cells.

### 3.3 GRP78 and cisplatin-associated senescence

GRP78 provides a more specific example of regulation of cisplatin-associated senescence. In ovarian cancer cell models, differences in GRP78 expression were associated with the susceptibility of cells to cisplatin-induced senescence, and forced GRP78 expression reduced the senescence response through mechanisms involving p53 and CDC2 signaling (32).

These findings support a role for GRP78 in modulating the threshold for cisplatin-associated growth arrest in the studied models. They do not establish GRP78 as a universal determinant of platinum resistance across OC.

### 3.4 Polyploidization and senescence-associated plasticity

Polyploid giant cancer cells (PGCCs) provide another example of treatment-associated cellular plasticity. In ovarian cancer cells exposed to docetaxel, PGCCs displayed senescence-associated characteristics together with expression of stem-cell-associated markers such as OCT4 and KLF4 (27). This observation indicates that treatment-associated polyploidization and senescence-like features can coexist with properties associated with cellular plasticity.

However, because the experimental treatment was docetaxel rather than platinum, these findings should not be interpreted as direct evidence that platinum-induced senescence escapes through PGCC formation. Rather, PGCC formation represents one experimentally described form of treatment-associated plasticity that may be relevant to persistence and recurrence.

## 4 The SASP and remodeling of the tumor microenvironment

The SASP is one of the major mechanisms through which senescent cells can influence neighboring cells. Its composition is highly heterogeneous, and different senescence-inducing stimuli can produce distinct secretory programs (8). Consequently, individual SASP factors should not be treated as interchangeable components of a single pathway.

In OC, secretory programs associated with treatment-induced senescence may affect tumor cells, fibroblasts, mesothelial cells, and immune populations. The consequences range from immune recruitment and inflammatory signaling to stromal remodeling and metastatic dissemination.

### 4.1 cGAS-dependent inflammatory signaling

The cGAS pathway has emerged as an important regulator of inflammatory signaling associated with cellular senescence. Apoptotic stress can promote mitochondrial DNA release during senescence and contribute to SASP formation (48). This work provides mechanistic context for the relationship between cellular damage, cytosolic DNA sensing, and senescence-associated inflammation.

OC-specific studies provide additional evidence that platinum treatment can promote inflammatory remodeling through senescence-associated mechanisms (22, 23). These findings support a connection between treatment-induced cellular stress and immune-active secretory programs, but the precise contribution of cGAS signaling may vary according to cellular and treatment context.

### 4.2 NAMPT-dependent metabolic adaptation

NAMPT has been implicated in a therapy-induced senescence-associated cancer stem-like population in OC (33). In cisplatin-treated ovarian cancer models, senescence-associated cells exhibited stem-related characteristics, including increased ALDH1A1 expression and CD133 positivity. NAMPT inhibition with FK866 reduced this population and enhanced the response to platinum treatment (33).

These findings identify NAMPT-dependent metabolism as a potential vulnerability of a defined treatment-associated population. They do not establish NAMPT as a universal regulator of senescence or the SASP.

### 4.3 TGFβ-enriched senescent secretome

A recent study provided evidence that a TGFβ-enriched senescent secretome contributes to resistance-associated phenotypes after platinum treatment across lung and ovarian cancer models (28). Mechanistic experiments established that cisplatin-derived secretome activates TGFBR1–AKT/mTOR signaling and promotes malignant phenotypes, with the strongest causal dissection performed in lung cancer models. In HGSOC, the study additionally demonstrated platinum-associated senescence and TGFβ/AKT/mTOR signatures in patient-derived material, supporting translational relevance but not proving that the same pathway alone drives clinical platinum resistance in OC.

This study is particularly relevant because it connects a defined platinum-associated senescent secretome to a tumor-promoting signaling pathway and provides both mechanistic preclinical evidence and supportive HGSOC patient-sample data (28). Nevertheless, TGFβ should not be presented as a universal SASP component or as an OC-specific clinical resistance biomarker. The appropriate interpretation is that a TGFβ-enriched secretome is one experimentally supported route by which platinum-induced senescence can promote tumor-supportive signaling.

### 4.4 IL-6 and stromal reprogramming

IL-6 has been implicated in drug resistance through formation of PGCCs and reprogramming of stromal fibroblasts in ovarian cancer-related experimental models (49). These findings suggest that inflammatory signaling can connect tumor-cell plasticity with changes in the stromal compartment.

The evidence does not justify treating IL-6 as a universal mediator of platinum resistance or as a defining component of every OC SASP. Its effects are better considered in relation to the specific cellular interactions and treatment conditions studied.

### 4.5 WNT16B and fibroblast–immune communication

WNT16B provides a distinct example of fibroblast-derived signaling. Ovarian fibroblast-derived WNT16B was reported to promote regulatory T-cell differentiation through β-catenin signaling in dendritic cells (41). This finding supports a role for fibroblast-derived WNT16B in immune regulation within the ovarian cancer microenvironment.

However, this study does not directly establish WNT16B as a mediator of platinum resistance. Its relevance to treatment response therefore lies in potential immune remodeling rather than a demonstrated tumor-cell-intrinsic resistance mechanism.

### 4.6 Extracellular vesicles and transmission of senescence-associated signals

Extracellular vesicles may provide another mechanism through which senescence-associated signals are transmitted between cells. YBX1 has been reported to regulate the loading of satellite II RNA into small extracellular vesicles and to promote senescence-associated phenotypes in recipient cells (42). Clinical dataset analyses in that study also identified YBX1 expression in cancer-associated stromal compartments.

These findings support the possibility that extracellular vesicles contribute to propagation of senescence-associated signals. However, the available evidence does not establish the YBX1–SATII pathway as a mechanism of platinum resistance in OC.

### 4.7 Chemotherapy-induced secretome and metastatic dissemination

Cole et al. now provide peer-reviewed evidence that a cisplatin-induced senescence-associated secretome can alter cholesterol homeostasis in neighboring HGSOC cells through an NAD+–SIRT1–SREBP-related metabolic mechanism, promoting cell detachment and metastatic dissemination (50). This work extends the functional consequences of chemotherapy-associated secretory signaling beyond classical inflammatory cytokines and links the cisplatin-induced secretome to a defined metastatic phenotype. Because the principal endpoint is metastatic dissemination rather than immune activation, immune evasion, or platinum resistance, the study should be interpreted as evidence for treatment-associated tumor remodeling rather than as direct proof of an immune mechanism.

### 4.8 SASP as an immunological rheostat

One way to reconcile apparently contradictory effects of the SASP is to treat it as an immunological rheostat rather than a fixed inflammatory program. Early or acute secretory responses can increase chemotaxis, antigen presentation, and cytotoxic lymphocyte engagement (10, 51), whereas prolonged exposure can reshape myeloid and lymphoid compartments and favor immune suppression. Senescent tumor cells can also acquire cell-intrinsic checkpoint protection, such as increased PD-L1, that limits T-cell-mediated clearance even when inflammatory cues remain present (15, 16). Recent work on the spatiotemporal dynamics of TIS similarly emphasizes that immune consequences may change with treatment duration and local microenvironmental conditions (51).

For OC, this temporal view is particularly relevant to TGFβ- and IL-6-associated signaling. A secretome that initially attracts immune cells may, with persistent exposure, promote fibroblast remodeling, suppress cytotoxic functions, or reinforce secondary senescence in neighboring compartments. Accordingly, the biologically relevant variable may be the duration and cellular source of SASP exposure rather than the presence of any single cytokine. This distinction is important when considering SASP-targeted interventions: indiscriminate suppression could attenuate beneficial immune recruitment, whereas selective blockade of a chronic, tumor-supportive program may preserve immune surveillance while limiting immune escape (8, 24, 28, 51).

## 5 Stromal and immune-cell senescence

Senescence in non-malignant cellular compartments may influence OC progression independently of senescence in tumor cells. These effects are particularly relevant in the peritoneal cavity, where fibroblasts, mesothelial cells, adipose-associated stromal populations, and immune cells interact with disseminated tumor cells.

### 5.1 Cancer-associated fibroblasts

PARP inhibitor exposure can induce senescence-associated changes in CAFs, and CAF-derived SASP has been implicated in resistance to PARP inhibition in OC models (37). Bepotastine reduced NF-κB-associated CAF SASP and increased sensitivity to PARP inhibitors in experimental models (37).

This provides evidence for a stromal mechanism of PARP inhibitor resistance, but not direct evidence that CAF senescence is a universal mechanism of platinum resistance. The distinction is important because PARP inhibitor resistance and platinum resistance overlap biologically but are not interchangeable clinical phenotypes.

Recent OC-specific studies further support the compartment-specific nature of stromal senescence. Perié et al. reported that senescent human ovarian fibroblasts undergo extracellular-matrix remodeling and develop an immune-modulatory, pro-inflammatory secretome; conditioned medium from these fibroblasts increased apoptosis resistance, migration, and invasion of ovarian cancer cells without increasing proliferation (52). More recently, Lori et al. showed that therapy-induced senescent ovarian fibroblasts can release increased glutamine, which is taken up by ovarian cancer cells and promotes invasion and stem-like features through SLC1A5 and a redox-dependent NRF2/ETS1 pathway (53). These findings are notable because they do not simply reproduce tumor-cell senescence. Instead, stromal senescence can reprogram the phenotype of surviving cancer cells through extracellular-matrix and metabolic signals, potentially changing their malignant behavior and the cellular context in which immune surveillance occurs. This provides a mechanistic bridge between TME senescence and tumor-cell persistence while also showing that relevant stromal outputs extend beyond classical cytokine SASP.

The compartment-specific evidence above suggests that stromal senescence may alter the fate of therapy-exposed tumor cells without necessarily converting them into senescent cells. In other words, stromal TIS can function as a paracrine modifier of cancer-cell survival, invasion, stemness, or immune visibility. This distinction is important when interpreting bulk tumor samples, because a high senescence signal may reflect a mixture of malignant TIS and treatment-aged stroma with different functional consequences (52, 53).

### 5.2 Mesothelial senescence and peritoneal colonization

The peritoneal mesothelium is a critical barrier and metastatic niche in OC. Senescent mesothelial matrices have been reported to enhance ovarian cancer-cell colonization (38). Earlier work similarly showed that senescent peritoneal mesothelium can create a permissive niche for ovarian cancer metastasis (39).

These findings suggest that senescence-associated remodeling of the mesothelial compartment can facilitate tumor-cell adhesion and colonization. They concern metastatic niche formation rather than direct tumor-cell platinum resistance.

Ovarian cancer cells can also influence stromal senescence-related phenotypes through paracrine signaling. Oxidative stress has been implicated in hepatocyte growth factor-dependent pro-senescence activity in ovarian cancer cells (54). Such interactions further emphasize that senescence in the ovarian cancer microenvironment is bidirectional rather than restricted to tumor cells.

### 5.3 Adipose-associated stromal senescence

The adipose-rich peritoneal environment is an important site of OC dissemination. In OC-bearing mice, dasatinib plus quercetin reduced senescence-associated changes in adipose tissue and, when combined with carboplatin or olaparib, reduced peritoneal and adipose tissue metastasis (40).

These results support a role for stromal senescence-associated states in metastatic progression and provide preclinical evidence for senolytic approaches. However, they do not establish adipose stromal senescence as a direct cause of tumor-cell platinum resistance.

### 5.4 Immune-cell senescence and clearance

Senescence-associated changes in immune cells may influence immune surveillance, inflammatory signaling, and clearance of damaged cells. Direct evidence connecting immune-cell senescence with platinum-resistant recurrence in OC remains limited.

SMARCA4 has recently been implicated in NK-cell-mediated recognition and elimination of senescent cells (29). In an immunocompetent ovarian cancer model, pharmacological/genetic targeting of SMARCA4 enhanced NK-mediated senescent-cell clearance and synergized with cisplatin to increase CD8+ T-cell and activated NK-cell infiltration (29). This finding links a senescence-regulatory pathway to immune-mediated clearance in OC rather than only to tumor-cell growth. Nevertheless, these findings remain preclinical and do not establish SMARCA4 inhibition as a clinically validated strategy for platinum resistance.

#### 5.4.1 T-cell senescence and T-cell exhaustion

T-cell dysfunction is central to the immunological consequences of senescence in cancer, but senescence and exhaustion should not be treated as interchangeable terms. Senescent T cells are generally defined by impaired proliferative capacity and altered stress responses, whereas exhausted T cells emerge from chronic antigen stimulation and develop distinct transcriptional and epigenetic programs with sustained inhibitory-receptor expression (24, 55). These states can overlap phenotypically but may differ in developmental origin, reversibility, metabolic dependencies, and response to checkpoint blockade. In OC, therapeutic pressure may create conditions in which tumor-cell TIS coexists with pre-existing or treatment-associated T-cell dysfunction. Accordingly, failure of immune clearance may result either from inadequate recognition of senescent tumor cells or from insufficient effector capacity in the responding T-cell compartment. OC-specific studies should therefore distinguish these states experimentally rather than inferring T-cell senescence from checkpoint expression alone (24, 55).

#### 5.4.2 NK-cell surveillance of senescent cells

NK cells provide a complementary route for immune-mediated senescent-cell clearance because their activity is not dependent on the same antigen-priming requirements as conventional CD8+ T-cell responses. Altered surface ligands and stress-associated phenotypes on senescent cells can facilitate NK recognition, while NK cells themselves are susceptible to age- and TME-associated functional decline (17, 25). The balance is therefore bidirectional: senescent-cell accumulation can impair immune homeostasis, whereas impaired NK competence can allow senescent populations to persist. Recent work further shows that SMARCA4 inhibition can enhance NK-mediated clearance of senescent cells and improve cisplatin-associated immune responses in an immunocompetent OC model (29). Thus, senescence-targeted therapy should consider not only the vulnerability of the target cell but also the cytotoxic capacity and metabolic fitness of the effector NK compartment.

#### 5.4.3 Myeloid and antigen-presenting compartments

Macrophages and dendritic cells can either facilitate clearance or contribute to persistence depending on their state and the signals they receive. Senescence-associated reprogramming of these populations may alter antigen processing, cytokine production, phagocytic activity, or suppressive signaling, thereby changing the probability that senescent tumor cells will be efficiently recognized and eliminated (17, 24). This distinction is mechanistically important because a senolytic strategy that removes senescent tumor cells may have limited immunological impact if APC function or myeloid polarization remains suppressive. Conversely, interventions that restore APC competence could amplify the consequences of TIS without directly increasing senolysis.

### 5.5 Cross-compartment coupling during treatment

Taken together, these observations suggest that senescence-associated biology in OC is a multicellular process rather than a tumor-cell or stromal event in isolation. A single therapeutic exposure may generate a mixed ecosystem in which malignant epithelial cells enter TIS while CAFs, mesothelial cells, or adipose-associated stromal cells undergo senescence or acquire SASP-like programs. These compartments can then feed back on one another. In OC, CAF-derived SASP has been linked to PARP inhibitor resistance (37), whereas senescent mesothelial matrices and adipose-associated senescence have been linked primarily to peritoneal colonization and metastatic dissemination (38–40). Together, these data are consistent with partially separable persistence/resistance and dissemination circuits.

The direction of causality remains incompletely resolved. Many available studies manipulate one compartment and measure a downstream tumor phenotype, making it difficult to determine whether stromal senescence is upstream of tumor-cell TIS, induced by tumor-cell SASP, or simply parallel to both. This distinction has direct therapeutic implications: eliminating senescent tumor cells may leave a pro-tumor stromal niche intact, whereas suppressing stromal SASP may remodel the niche without removing relapse-competent tumor cells. Time-resolved single-cell and spatial studies that track the same cell populations before treatment, during TIS, and after treatment withdrawal will be needed to resolve these relationships.

### 5.6 Aging, immunosenescence, and the threshold for immune clearance

Aging adds another layer of heterogeneity because the generation of senescent cells and the capacity to clear them are not independent processes (**Table 2**). Age-associated immunosenescence can reduce the proliferative and cytotoxic capacity of T and NK cells and alter myeloid-cell function, potentially shifting the balance away from effective surveillance. Conversely, persistent senescent-cell accumulation can contribute to chronic inflammatory signaling that further impairs immune homeostasis. Recent cancer-immunology literature therefore frames senescence and immunosenescence as interacting components of tumor immune escape rather than as separate phenomena (17, 25).

**Table 2:** Immunological consequences of senescence-associated states in ovarian cancer.

Senescence-associated compartment	Immune interaction	Potential functional consequence	Evidence context
Tumor-cell TIS	MHC-I/interferon-associated activation; DC and CD8+ T-cell engagement	Immune recognition and clearance	Conserved mechanism; not OC-specific (10)
HR-deficient HGSOC with TIS	cGAS/STING-associated SASP; CD8+ T-cell and NK-cell infiltration	Platinum sensitivity and increased response to immune checkpoint blockade	Primary OC evidence in immunocompetent models (23)
Persistent tumor-cell TIS	Chronic SASP; TGFβ/IL-6 signaling; immune-evasion checkpoints	Immune suppression, persistence, or relapse	OC and mechanism-specific evidence (15, 28, 49, 51)
Senescent CAFs	SASP-driven paracrine signaling; NF-κB activation	Stromal remodeling and PARPi resistance	Direct OC evidence; PARPi-specific (37)
Senescent ovarian fibroblasts	ECM remodeling; immune-modulatory SASP; stromal-derived glutamine	Cancer-cell apoptosis resistance, invasion, and stem-like traits	Recent OC-specific primary evidence (52, 53)
Senescent mesothelial/adipose stroma	Matrix and niche remodeling	Peritoneal colonization and dissemination	OC metastasis models (38–40)
Senescent T cells	Reduced proliferative capacity and altered effector function	Impaired immune surveillance and reduced clearance capacity	OC-focused and broader immune-cell senescence evidence (17, 24)
Exhausted T cells	Chronic antigen stimulation and sustained inhibitory-receptor signaling	Persistent T-cell dysfunction and potentially reduced ICI responsiveness	Distinct from T-cell senescence; broader cancer immunology (55)
Senescent/dysfunctional NK cells	Reduced cytotoxic competence; altered recognition of stressed/senescent targets	Reduced senescent-cell clearance and immune surveillance	General cancer evidence with OC implications (17, 25, 29)
Senescent macrophage/DC compartments	Altered antigen processing, cytokine production, and suppressive signaling	Weakened immune priming or establishment of an immunosuppressive niche	OC-focused synthesis and broader immune-cell senescence evidence (17, 24)
TIS-associated PD-L1 upregulation	PD-1/PD-L1 checkpoint signaling; reduced CTL susceptibility	Immune evasion and persistence of therapy-induced senescent cells	Preclinical cancer evidence; not OC-specific (15)
TIS-associated CD47 expression	CD47-SIRPα “don’t-eat-me” signaling to myeloid cells	Potential evasion of innate immune clearance	Emerging review-level concept (14)

APC, antigen-presenting cell; CAF, cancer-associated fibroblast; CD8+ T cell, cytotoxic T lymphocyte; DC, dendritic cell; HGSOC, high-grade serous ovarian cancer; NK, natural killer; OC, ovarian cancer; PARPi, PARP inhibitor; SASP, senescence-associated secretory phenotype; TIS, therapy-induced senescence.

For OC, this framework also helps explain why a senescence marker detected after therapy cannot be interpreted without knowing the immune context. A tumor enriched for senescent cells but surrounded by competent DCs and cytotoxic lymphocytes may follow a different trajectory from one in which senescent tumor or stromal populations coexist with dysfunctional T cells, impaired NK surveillance, or suppressive myeloid populations. The clinically relevant unit may therefore be the senescent cell together with its immune “clearance capacity,” rather than senescence burden alone (24, 25, 55).

## 6 Therapeutic strategies targeting defined senescence-associated states

The heterogeneity of senescence has important implications for therapy (**Figure 2**). The available evidence does not support a single strategy of either inducing or eliminating senescence. Instead, therapeutic approaches should be matched to the biological state that contributes to treatment response or disease progression.

**Figure 2:**
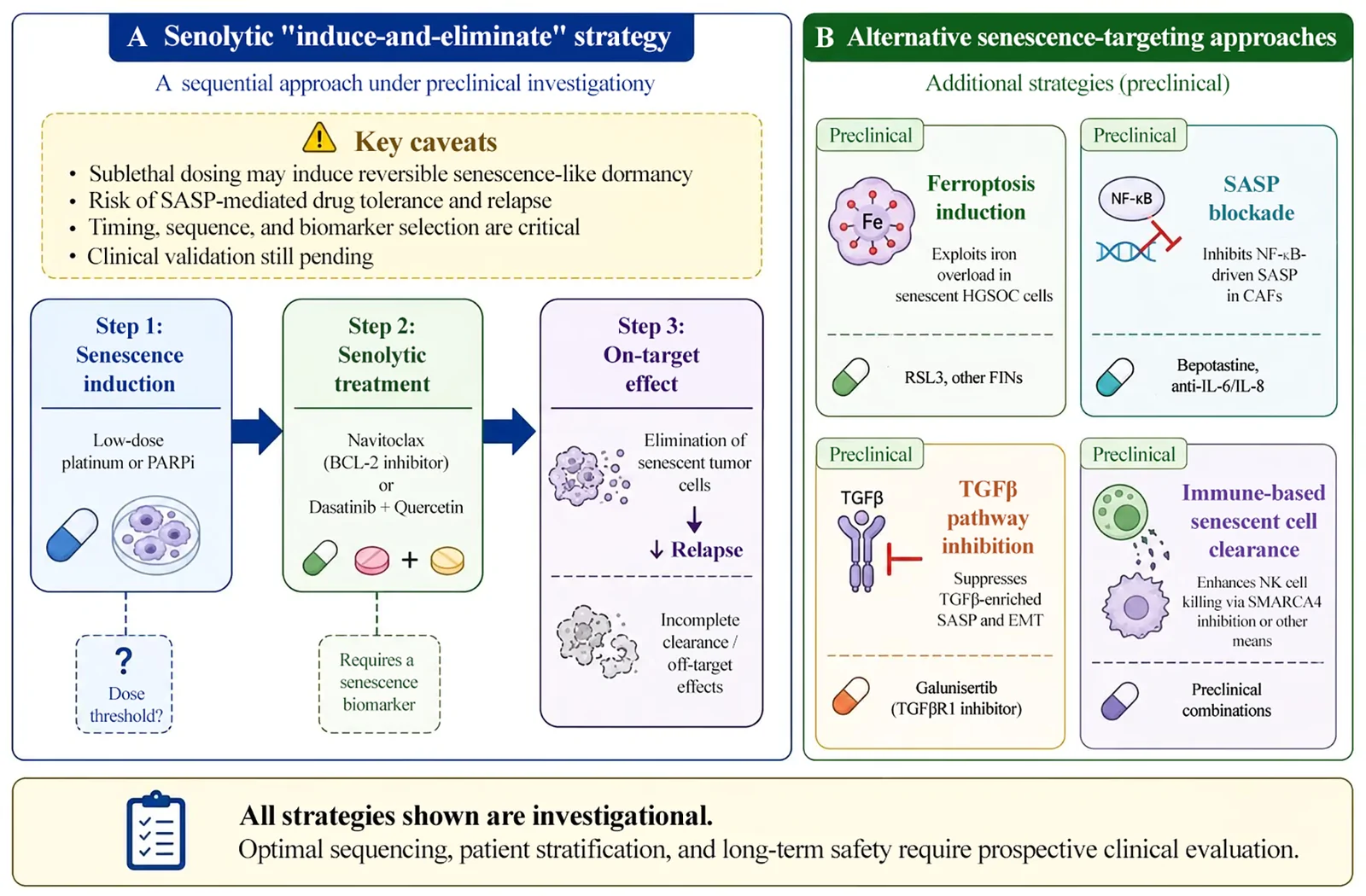
State-specific therapeutic targeting of senescence-associated states in ovarian cancer. **(A)** Senolytic “induce-and-eliminate” strategy, showing senescence induction, senolytic treatment, and the intended on-target effect, together with key caveats. In HGSOC models, olaparib-induced reversible senescence-like cells in OV4453 and OV1946 were sensitive to ABT-263. **(B)** Alternative senescence-targeting approaches, including ferroptosis induction, SASP blockade, TGF-β pathway inhibition, and immune-based clearance. Immune-directed strategies remain largely preclinical in OC (10, 15, 23, 26–29).

### 6.1 Senolytic approaches

Senolytic therapy aims to eliminate cells that depend on specific survival pathways associated with senescence. In OC models, dasatinib plus quercetin reduced senescence-associated changes in adipose tissue and, when combined with carboplatin or olaparib, reduced peritoneal and adipose tissue metastasis (40). These findings support the possibility that stromal senescence-associated populations can be therapeutically targeted.

The timing of senolytic treatment may be particularly important. PARP inhibition can induce a reversible senescence-associated state in OC cells, with cell-cycle re-entry observed after drug withdrawal in experimental models (26). This provides a rationale for sequential treatment strategies in which therapy-induced persistent cells are targeted after the initial cytotoxic or cytostatic treatment.

A particularly informative OC example comes from the PARP inhibitor–senolytic combination studied by Fleury et al. In the HGSOC models OV4453 and OV1946, olaparib induced a senescence-like, reversible state associated with increased BCL-XL, and ABT-263 (navitoclax) showed preferential activity when cells were in the treatment-associated senescence-like state (26). The investigators also demonstrated activity in HGSOC xenograft models and found that sensitivity to ABT-263 diminished after senescence reversal in models that more readily regained proliferation (26). This observation is important because it links three otherwise separate concepts—reversibility, a defined survival dependency, and senolytic vulnerability—and provides a mechanistic rationale for sequential treatment rather than indiscriminate senolysis. The broader 2020 Wang et al. review places this finding within the emerging framework of therapy-induced senescence as a targetable but heterogeneous cancer-cell state (34). Because the evidence is PARP inhibitor based rather than platinum specific, it should be presented as proof-of-concept for senescence-targeted therapy in OC, not as evidence that ABT-263 reverses platinum resistance.

At present, however, the optimal timing, selectivity, and toxicity of such strategies remain unresolved. Senolysis may also affect normal senescent cells with physiological roles in tissue repair and homeostasis.

This translational caution is reinforced by recent critical appraisal of senolytic therapy, which emphasizes that many efficacy claims still depend on model-specific definitions of senescence, treatment timing, and drug response. Senolytic activity against a marker-positive population should therefore be distinguished from selective elimination of the relapse-competent cells that drive clinically meaningful recurrence (56).

### 6.2 SASP modulation

An alternative approach is to suppress harmful secretory activity without necessarily eliminating the senescent cell. The therapeutic rationale is particularly relevant when the growth-arrested state itself is tumor suppressive but its secretome promotes tumor progression.

TGFBR1 inhibition has been investigated in the context of a TGFβ-enriched senescent secretome associated with platinum-treatment resistance (28). CAF-associated SASP can also be modulated pharmacologically with bepotastine in experimental PARP inhibitor-resistance models (37). NAMPT inhibition represents a different strategy, targeting a metabolic dependency associated with a therapy-induced senescence-associated cancer stem-like population (33).

These approaches should not be considered interchangeable “SASP inhibitors.” They target distinct pathways and potentially different cellular populations.

### 6.3 Ferroptosis-based vulnerabilities

Persistent HGSOC cells may acquire altered iron metabolism that creates a vulnerability to ferroptosis. Treatment-associated persistent HGSOC cells have been reported to exhibit dysregulated iron homeostasis and increased sensitivity to ferroptosis-inducing approaches such as RSL3 (21). Ferroptosis is characterized by iron-dependent lipid peroxidation and oxidative membrane damage and therefore provides a mechanistically distinct form of cell death (57).

This strategy should not, however, be equated with senolysis. Ferroptotic agents may affect multiple cell populations within the TME, and the consequences of lipid peroxidation for immune and stromal cells remain important considerations.

CircASH1L has also been reported to inhibit ferroptosis and promote cisplatin resistance through the miR-515-5p/CDCA7/RRM2 axis in ovarian cancer cells (58). This study supports ferroptosis-related mechanisms as part of cisplatin resistance biology, but it does not establish CircASH1L as a specific regulator of senescence.

### 6.4 Restoring tumor-suppressive senescence

Not all therapeutic strategies should eliminate senescent cells. In selected resistant models, restoring a tumor-suppressive growth-arrest program may improve treatment sensitivity.

Aurora-A/SOX8/FOXK1 signaling suppresses senescence and promotes chemoresistance, whereas the NACC1/GADD45GIP1 axis has been linked to cisplatin resistance through altered senescence (30, 31). These findings support restoration of growth arrest in selected tumor-cell contexts, but the evidence remains model-specific.

PTEN has also been reported to induce ovarian cancer cell senescence through a p21-associated mechanism (59). EZH2 interference can reverse cisplatin resistance, but the reported mechanism centers on autophagy rather than a demonstrated senescence pathway (60).

Restoring senescence and eliminating senescent cells are therefore distinct strategies. The appropriate choice depends on whether treatment failure reflects escape from arrest, persistence of a vulnerable senescent population, or a stem-like residual state.

ALDH1A1 inhibition has been reported to reduce platinum-induced senescence and stemness in ovarian cancer models (61), supporting a state-specific approach to treatment-persistent populations.

Selective CDK2 inhibition can induce arrest, apoptosis, and senescence-associated phenotypes in CCNE1-amplified OVCAR3 cells (62). Its relevance to platinum response remains unresolved.

### 6.5 Immunosenolysis and combination with immunotherapy

Immunosenolysis remains an emerging strategy rather than an established treatment (63). NK cells and engineered CAR-T/CAR-NK cells may provide immune-mediated routes to remove senescent cells, but current evidence is largely preclinical. A recent Frontiers in Immunology review discusses CAR-T/NK-based approaches and highlights target heterogeneity and apoptotic priming, but does not provide clinical efficacy data in OC (64).

In OC, direct preclinical evidence comes from immunocompetent HGSOC models. Senescence-prone HR-deficient tumors showed enhanced platinum responsiveness, while a restricted cGAS/STING-associated SASP increased immune infiltration and sensitized tumors to checkpoint blockade (23). SMARCA4 inhibition also enhanced NK-mediated clearance of senescent cells and synergized with cisplatin in an immunocompetent ovarian cancer model (29). These findings support immune-mediated clearance of senescent cells in OC, but they do not establish a clinically validated immunosenolytic therapy.

For OC, the relevant question is which immune state is present when senescence-targeted therapy is given. Functional antigen presentation and CD8+ T/NK-cell activity may permit clearance, whereas chronic SASP, T-cell dysfunction, or myeloid tolerance may favor persistence (14, 24, 25, 51, 55, 64).

No senolytic or immunosenolytic regimen has yet been clinically validated as a biomarker-guided treatment for OC, and the optimal balance between eliminating senescent targets and preserving beneficial immune surveillance remains unresolved. This uncertainty is particularly relevant to immune-combination strategies because broad depletion of senescent cells could remove both tumor-supportive and immunostimulatory populations.

An additional strategy is to remove immune-evasion signals from senescent tumor cells. CD47 has recently been proposed as a molecular bridge between TIS and innate immune tolerance, providing a rationale for CD47/SIRPα-directed approaches that could facilitate macrophage-mediated clearance (14). This links the senescent state to an established innate immune-checkpoint axis. Nevertheless, current evidence is primarily based on broader cancer models and mechanistic syntheses rather than OC-specific therapeutic trials; CD47 should therefore be considered a candidate immunosenolytic checkpoint, not a validated OC biomarker or treatment.

## 7 Biomarkers and clinical translation

For clinical translation, biomarker development should capture both the senescent state and the capacity of the immune system to respond to it. Candidate measurements should combine tumor-cell senescence markers with immune-visibility features such as MHC-I, interferon-associated signaling, and PD-L1 or CD47 expression (10, 14, 15). Immune-cell composition and functional CD8+ T-cell or NK-cell activity should be assessed in parallel (17, 24, 25).

The clinical development of senescence-targeted therapies requires reliable identification of the relevant cellular state. No single marker is sufficient to define senescence across all tissues, experimental systems, or stages of disease.

SA-β-gal activity, p16, p21, persistent DNA-damage signaling, chromatin alterations, and SASP components can provide complementary information, but each marker has limitations (6–8). A treatment-associated increase in SA-β-gal or p16 should therefore not be interpreted as definitive evidence of functional senescence without additional evidence of sustained proliferative arrest and, where appropriate, characteristic secretory or metabolic changes.

The same issue applies to transcriptomic senescence signatures. Gene-expression programs identified in HGSOC models treated with chemotherapy may provide useful starting points for biomarker development (21, 40), but their clinical interpretation requires validation across independent cohorts and treatment settings.

An important distinction is that between prognostic and predictive biomarkers. A senescence-associated feature may correlate with survival or recurrence without identifying patients who will benefit from senolysis, SASP inhibition, or another senescence-directed treatment. A clinically useful therapeutic biomarker should ideally identify the specific cell population and state that is causally related to treatment persistence or disease progression.

A further challenge is temporal resolution. The same marker may identify different states at different time points. p21 or SA-β-gal positivity shortly after treatment may indicate an adaptive arrest. After prolonged drug withdrawal, the same signal may instead reflect persistent senescence, a reversible persister state, or residual stromal senescence. A useful biomarker panel should therefore combine cell identity, evidence of sustained arrest, DNA-damage persistence, secretory phenotype, and functional recovery after treatment withdrawal.

For clinical translation, the major gap is not the absence of senescence markers but the lack of prospectively validated immune-senescence biomarkers linked to treatment benefit. Current evidence in OC remains dominated by preclinical models, retrospective tissue analyses, and disease-specific reviews. Prospective studies should therefore pair spatially resolved senescence measurements with immune-state profiling before and after therapy, including APC activation, CD8+ T-cell function, NK-cell competence, and checkpoint-ligand expression. Such studies would directly address whether senescence burden predicts immune clearance, immune escape, or response to a senescence-directed intervention (17, 24, 51).

For OC, this will likely require integration of molecular measurements with spatial and cellular information. A bulk tumor sample containing senescent tumor cells, CAFs, and mesothelial or immune populations may generate a composite signal that cannot be assigned to a single cellular compartment.

## 8 Outstanding questions and future directions

Several questions remain unresolved.

### 8.1 Is therapy-induced senescence truly irreversible?

This question is not merely semantic. If a residual tumor cell can re-enter the cell cycle after drug withdrawal, then a short-term increase in senescence markers may represent a transient survival strategy rather than durable tumor suppression. Conversely, persistent senescence may reduce immediate tumor burden while increasing long-term inflammatory or stromal signaling. Serial sampling and functional “washout” assays are therefore essential to distinguish durable arrest from reversible TIS-associated dormancy.

The distinction between irreversible senescence, reversible senescence-like arrest, quiescence, and drug-tolerant persister states remains critical. PARP inhibitor-treated OC models have demonstrated reversible senescence-associated states (26), raising the possibility that some residual cells can re-enter the cell cycle after treatment withdrawal. Future studies should define these states functionally rather than relying solely on senescence markers.

### 8.2 Which cellular compartment is responsible for the observed phenotype?

Tumor cells, CAFs, mesothelial cells, adipose-associated stromal cells, and immune populations can all exhibit senescence-associated changes. Their effects are unlikely to be equivalent. Single-cell and spatial approaches may help identify which cell populations are induced by treatment and which subsequently influence tumor persistence or metastasis.

### 8.3 Which SASP components are functionally important?

The SASP should not be treated as a uniform inflammatory program. TGFβ-enriched secretomes, IL-6-associated stromal remodeling, WNT16B-mediated immune regulation, and extracellular-vesicle-associated signaling represent distinct mechanisms (28, 41, 42, 49). Future studies should determine which secreted factors are causal rather than simply associated with senescence.

### 8.4 How specific is the relationship to platinum resistance?

This question is particularly important. Evidence for PARP inhibitor resistance, paclitaxel resistance, metastatic dissemination, or immune remodeling should not automatically be extrapolated to platinum resistance. The strongest evidence for a platinum-associated relationship currently comes from defined cisplatin/platinum models involving pathways such as Aurora-A/SOX8/FOXK1, NACC1/GADD45GIP1, NAMPT-associated persistent populations, and TGFβ-enriched senescent secretomes (28, 30, 31, 33).

### 8.5 Can senescence be targeted without disrupting normal tissue homeostasis?

Senescent cells also participate in physiological processes, including wound healing and tissue remodeling. Broad senolytic strategies may therefore produce unintended effects. Cell-type-selective targeting or treatment schedules based on transient vulnerabilities may offer greater therapeutic specificity.

### 8.6 How should senescence-directed therapies be sequenced?

The biological state induced by chemotherapy may change over time. Immediate senolysis, delayed senolysis, SASP inhibition, or restoration of growth arrest could have different effects depending on when they are administered. Sequential treatment studies should therefore incorporate longitudinal assessment of cellular state rather than assuming that senescence remains static after chemotherapy.

### 8.7 Can patient-derived models resolve this heterogeneity?

Patient-derived organoids, ex vivo cultures, immune-competent models, humanized systems, and spatial or single-cell analyses may help identify clinically relevant senescence-associated populations. Such approaches could also clarify whether a senescence signature reflects residual tumor cells, stromal remodeling, or both.

### 8.8 When does TIS become immune priming rather than immune escape?

A central translational question is whether the immune-stimulatory phase of TIS can be prolonged or converted into effective tumor clearance before chronic SASP signaling establishes immune suppression. Longitudinal studies should jointly measure senescent tumor burden, APC activation, CD8+ T-cell state, NK-cell activity, and SASP composition rather than measuring senescence in isolation. This would allow identification of a therapeutic window in which senolysis or immune stimulation is most likely to reinforce, rather than disrupt, antitumor immunity (10, 25, 51).

### 8.9 How should senescence be distinguished from T-cell exhaustion in OC?

This distinction will become increasingly important as senescence-targeted interventions are combined with immunotherapy. Exhausted and senescent T cells can share checkpoint expression and functional impairment, but they differ in developmental cues and may differ in reversibility, transcriptional state, metabolic dependencies, and response to checkpoint blockade (24, 55). Future OC studies should therefore combine phenotypic markers with functional assays of proliferation, cytokine production, cytotoxicity, and recovery after stimulation or drug withdrawal. This distinction is necessary to determine whether a failed immune response reflects a senescent effector compartment, an exhausted effector compartment, or both.

### 8.10 What evidence would establish causality between senescence and immune-mediated treatment outcome?

A recurring limitation across the literature is that senescence, immune remodeling, and treatment resistance are often measured in parallel rather than causally connected. Stronger evidence would require state-specific perturbation and rescue experiments, separation of tumor-cell and stromal compartments, depletion or functional inhibition of defined immune populations, and longitudinal sampling before treatment, during TIS, and after treatment withdrawal. In OC models, combining these approaches with single-cell or spatial profiling and functional assessment of tumor-cell regrowth would help determine whether immune clearance is eliminating the relapse-competent population or merely altering its abundance. This distinction is essential for deciding whether a senescence-directed intervention should enhance immune clearance, suppress a harmful secretome, or selectively eliminate residual tumor cells (14, 24).

A second translational requirement is prospective clinical sampling. Paired pretreatment and on-treatment specimens should ideally measure tumor-cell senescence, stromal senescence, immune-cell state, and their spatial relationships within the same patient. This would help distinguish a biomarker that merely tracks treatment exposure from one that predicts immune clearance or identifies a residual population amenable to senolysis or immunotherapy. Such studies are particularly needed in OC, where prospective evidence linking senescence to immune-treatment benefit remains limited (17, 51).

## 9 Conclusions

Cellular senescence in OC is best understood as a context-dependent treatment-associated state rather than a uniformly tumor-suppressive or tumor-promoting process.

Platinum treatment can induce senescence-associated phenotypes in ovarian cancer cells and remodel the tumor microenvironment. Under selected conditions, this response can contribute to tumor control through sustained growth arrest and immune activation. In other settings, persistent or reversible senescence-associated states may coexist with stem-like properties, metabolic adaptations, polyploidization, or secretory programs that support tumor persistence.

The available evidence does not support a single pathway in which senescence universally causes platinum resistance. Instead, different mechanisms operate in different treatment and cellular contexts. Aurora-A/SOX8/FOXK1 and NACC1/GADD45GIP1 provide evidence that suppression or alteration of senescence can contribute to cisplatin resistance in specific tumor-cell models (30, 31). GRP78 and NAMPT provide additional examples of regulation of cisplatin-associated senescence and therapy-induced senescence-associated populations (32, 33). By contrast, PART1/PHB2 and miR-433 link senescence-associated states to PARP inhibitor or paclitaxel resistance, respectively, and should not be used as direct evidence of platinum resistance (35, 36).

The SASP adds another layer of complexity. TGFβ-enriched secretomes provide particularly relevant evidence for a platinum-associated mechanism (28), whereas IL-6, WNT16B, extracellular-vesicle signaling, and chemotherapy-associated metabolic remodeling represent distinct pathways with effects on stromal remodeling, immune regulation, or metastatic dissemination (41, 42, 49, 50). These mechanisms should not be collapsed into a single universal SASP program.

Senescence in stromal populations may likewise influence OC progression without directly causing tumor-cell platinum resistance. CAF-associated SASP has been linked to PARP inhibitor resistance (37), while senescent mesothelial and adipose-associated stromal compartments have primarily been associated with metastatic niche formation and peritoneal dissemination (38–40).

These observations support a shift from nonspecific manipulation of senescence toward precision targeting of defined senescence-associated states. Senolysis, SASP modulation, restoration of tumor-suppressive growth arrest, immune-mediated clearance, and exploitation of metabolic vulnerabilities such as ferroptosis are promising approaches, but most remain preclinical or investigational.

The central challenge is therefore not to classify senescence as beneficial or detrimental in the abstract, but to define which cell enters a senescence-associated state, after which treatment, for how long, and with what immune and secretory characteristics. In an immunology-focused setting, this means determining whether senescent tumor cells are efficiently recognized and cleared or persist when immune effector function is impaired.
